# Supplementary material for: The interactome of intact mitochondria by cross-linking mass spectrometry provides evidence for coexisting respiratory supercomplexes
Source: Mol Cell Proteomics. 2017 Dec 8;17(2):216–32. doi: 10.1074/mcp.RA117.000470 (PMC5795388; doi:10.1074/mcp.RA117.000470)
Supplement: Supplemental Data [file supp_RA117.000470_133922_0_supp_23978_fzffwf.zip › spectra_annotation/mito_DR_spectra_annotation/229-2-7-1-9-1.pdf]

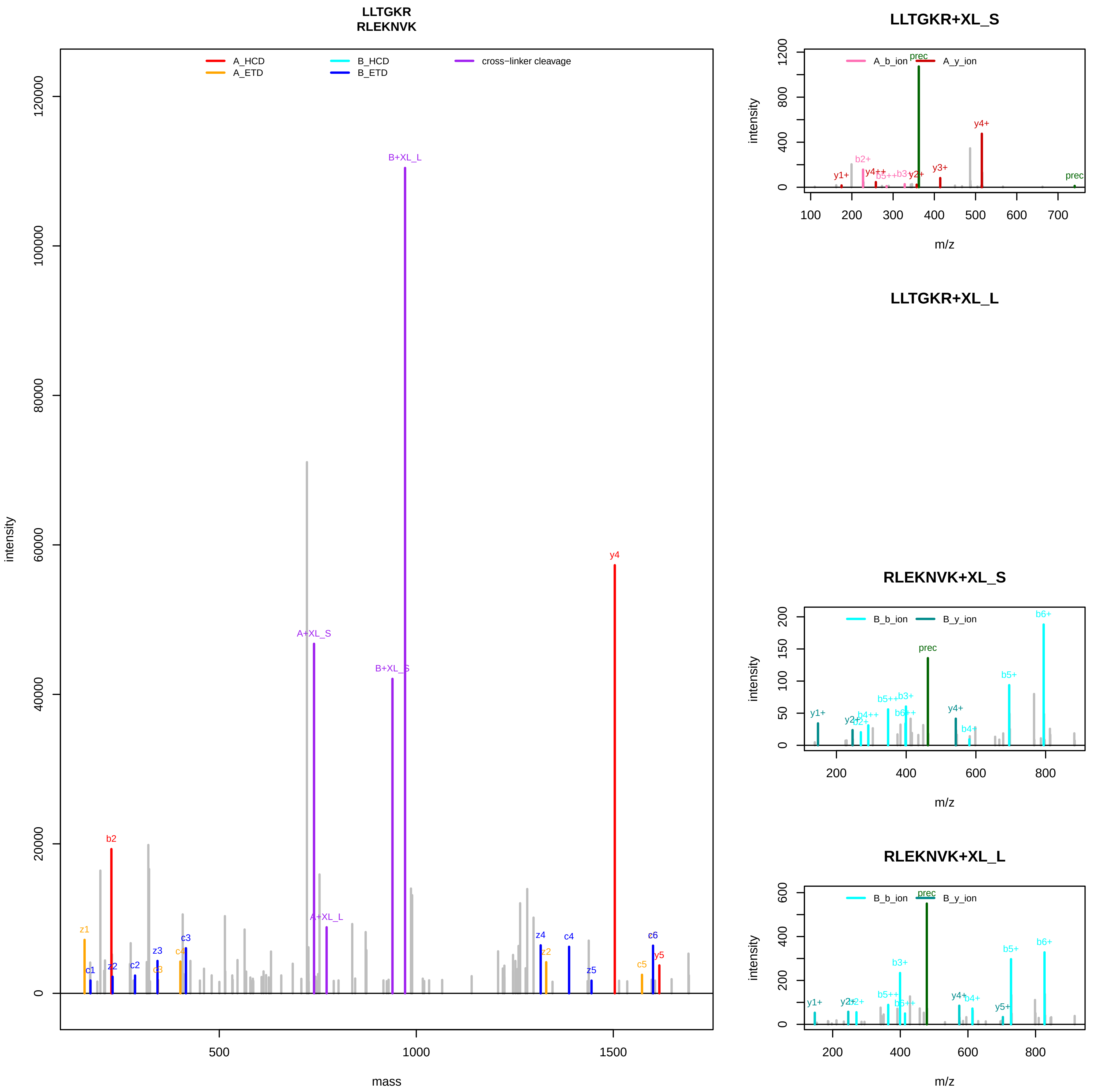

LLTGKR
RLEKNVK
A_HCD
A_ETD
B_HCD
B_ETD
cross−linker cleavage
intensity
mass
0
20000
40000
60000
80000
100000
120000
500
1000
1500
B+XL_L
A+XL_S
B+XL_S
A+XL_L
y4
b2
y5
z1
c1
z2
c2
z3
c3
c4
z4
z2
c4
z5
c5
c6
LLTGKR+XL_S
A_b_ion
A_y_ion
prec
y4+
b2+
y1+
y4++
b5++
b3+
y2+
y3+
m/z
LLTGKR+XL_L
RLEKNVK+XL_S
B_b_ion
B_y_ion
b6+
b5+
y1+
y2+
b2+
b4++
b5++
b3+
b6++
y4+
b4+
RLEKNVK+XL_L
y5+
